# Tau mutation S356T in the three repeat isoform leads to microtubule dysfunction and promotes prion-like seeded aggregation

**DOI:** 10.3389/fnins.2023.1181804

**Published:** 2023-05-25

**Authors:** Yuxing Xia, Brach M. Bell, Justin D. Kim, Benoit I. Giasson

**Affiliations:** ^1^Department of Neuroscience, College of Medicine, University of Florida, Gainesville, FL, United States; ^2^Center for Translational Research in Neurodegenerative Disease, College of Medicine, University of Florida, Gainesville, FL, United States; ^3^McKnight Brain Institute, College of Medicine, University of Florida, Gainesville, FL, United States; ^4^Department of Internal Medicine, College of Medicine, University of Florida, Gainesville, FL, United States

**Keywords:** tau protein, aggregation, microtubule, frontotemporal dementia, Alzheimer’s disease

## Abstract

Tauopathies are a group of neurodegenerative diseases, which include frontotemporal dementia (FTD) and Alzheimer’s disease (AD), broadly defined by the development of tau brain aggregates. Both missense and splicing tau mutations can directly cause early onset FTD. Tau protein is a microtubule-associated protein that stabilizes and regulates microtubules, but this function can be disrupted in disease states. One contributing factor is the balance of different tau isoforms, which can be categorized into either three repeat (3R) or four repeat (4R) isoforms based on the number of microtubule-binding repeats that are expressed. Imbalance of 3R and 4R isoforms in either direction can cause FTD and neurodegeneration. There is also increasing evidence that 3R tauopathies such as Pick’s disease form tau aggregates predominantly comprised of 3R isoforms and these can present differently from 4R and mixed 3R/4R tauopathies. In this study, multiple mutations in 3R tau were assessed for MT binding properties and prion-like aggregation propensity. Different missense tau mutations showed varying effects on MT binding depending on molecular location and properties. Of the mutations that were surveyed, S356T tau is uniquely capable of prion-like seeded aggregation and forms extensive Thioflavin positive aggregates. This unique prion-like tau strain will be useful to model 3R tau aggregation and will contribute to the understanding of diverse presentations of different tauopathies.

## 1. Introduction

Tau is a microtubule-associated protein that is important for microtubule (MT) assembly, regulation, and stability in neurons (Wang and Mandelkow, 2016). In neurodegenerative diseases termed tauopathies, tau protein can form toxic brain aggregates. These disorders include frontotemporal dementia (FTD), Pick’s disease (PiD), progressive supranuclear palsy (PSP), corticobasal degeneration (CBD), argyrophilic grain disease (AGD), Alzheimer’s disease (AD), and chronic traumatic encephalopathy (CTE; Zhang et al., 2022). Missense tau mutations can directly cause familial forms of FTD (Hutton et al., 1998; Strang et al., 2019) and the progression of tau pathology is associated with different tau conformations and prion-like strains that can spread between cell types and in various brain regions (Ayers et al., 2018; Chung et al., 2019).

**Figure 1:**
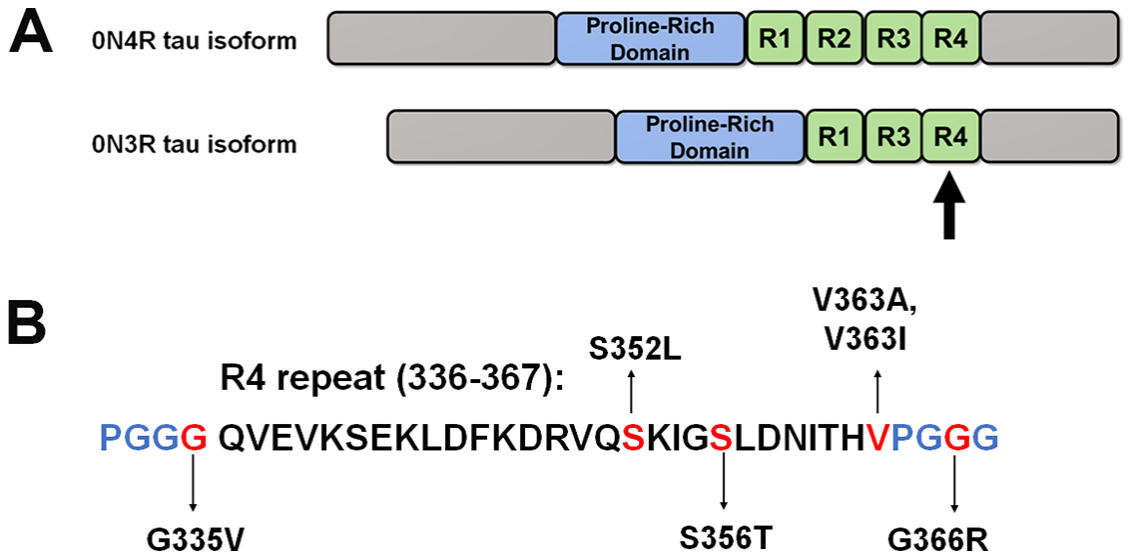
Schematic of 0N4R and 0N3R tau isoforms and tau mutants investigated in this study. **(A)** This diagram depicts the overall structure of 0N4R and 0N3R tau isoforms with major domains. Within the MT binding region, R1 to R4 represent the MT binding repeats. **(B)** Specific amino acid sequence of the fourth repeat is shown. The positions of tau mutations are highlighted in red. Blue letters represent the other amino acids that comprise the PGGG motifs within each MT repeat.

Tauopathies can further be classified based on the relative balance of different tau isoforms. Physiologically, tau protein is alternatively spliced into six different isoforms, which can be grouped into either three or four microtubule-associated binding repeats (3R or 4R) (Figure 1; Goedert and Jakes, 1990; Liu and Gong, 2008). Disease-associated tau inclusions in tauopathies can be comprised of tau polymerized into filaments composed of either 3R isoforms, 4R isoforms, or a mix of both 3R and 4R isoforms (Zhang et al., 2022). Tau aggregates assembled from 3R or 4R isoforms have structurally distinct conformations and are related to the development of different types of tauopathies (Scheres et al., 2020; Vaquer-Alicea et al., 2021); however, many common tauopathies including AD and FTD have tau aggregates composed of both 3R and 4R isoforms (Iqbal et al., 2016; Zhang et al., 2022).

These different tau isoforms have altered abilities to regulate MT functions. For example, 4R tau isoforms bind more tightly to MTs and promote both MT growth and stability better than 3R tau isoforms (Goedert and Jakes, 1990; Goode et al., 2000; Panda et al., 2003). There are more types of 4R predominant tauopathies (e.g., PSP, CBD, GGT, AGD) than 3R ones (e.g., Pick’s disease; Zhang et al., 2022). WT 3R tau isoforms have also been postulated to inhibit the formation of 4R tau filaments, and may naturally be less prone to tau aggregate formation (Adams et al., 2010).

Recently, our group reported significant differences in MT binding and aggregation for tau mutations Q336H and Q336R in the context of 3R and 4R tau isoforms (Xia et al., 2021b). These findings warrant further investigation into the properties of other tau mutations within 3R and 4R tau isoforms that may elucidate new pathogenic mechanisms. In this study, we selected tau mutations located close to the third and fourth repeats within the microtubule-binding region (MTBR) and assessed their properties in MT binding and aggregation propensity. The fourth repeat is an important region that forms the core of tau aggregates and is common to both 3R and 4R tau isoforms (Fitzpatrick et al., 2017).

## 2. Materials and methods

### 2.1. Purification of K19 tau protein

The K19 tau fragment, which corresponds to the MT binding region of 3R tau (Q244 to E372 as numbered according to the sequence of the 2N4R tau isoform but without the R2 repeat), was expressed in BL21 (DE3)/RIL *Escherichia coli* (Agilent Technologies, Santa Clara, CA) using the pRK172 plasmid. Recombinant K19 tau protein was purified as previously described (Xia et al., 2019, 2021b).

### 2.2. Preparation of K19 tau amyloid seeds

Purified K19 tau protein was diluted to 1 mg/mL in sterile phosphate-buffered saline (PBS) and 50 μM of heparin (Fisher Scientific, Waltham, MA). The resulting mixture was incubated by shaking at 1050 RPM and 37°C for 2 days. K114 or thioflavin T assays (Crystal et al., 2003) were used to confirm polymerization into amyloid. K19 tau fibrils were centrifuged at 100,000 g for 30 min and re-dissolved in PBS to remove heparin. The K19 fibrils were water bath sonicated for 1 h to produce short fibrils (Waxman and Giasson, 2011; Xia et al., 2019).

### 2.3. Mammalian expression plasmids and site-directed mutagenesis

The 0N3R human tau cDNA isoform was cloned into mammalian expression vector pcDNA3.1 (+). The different missense *MAPT* mutations studies in 0N3R human tau were created with QuikChange site-directed mutagenesis (Agilent Technologies, Santa Clara, CA) using mutation-specific oligonucleotides. All mutations and the entire cDNA tau sequences were confirmed by Sanger sequencing at Genewiz (South Plainfield, NJ).

### 2.4. Cell culture and calcium phosphate transfection

HEK293T cells were cultured at 37°C and 5% CO_2_ in Dulbecco’s modified Eagle’s media and 10% fetal bovine serum (FBS) with added antibiotics (100 units/mL penicillin, 100 μg/mL streptomycin). Different plasmids expressing either WT tau or tau mutants were transfected into HEK293T cells by calcium phosphate precipitation (Xia et al., 2019, 2021b). Briefly, cells were split into 12-well plates at 20–40% confluency. For each well, 1.5 μg of DNA was mixed with 18.75 μL of 0.25 M CaCl_2_. This mixture was added to an equal amount of 2X BES buffer (50 mM BES, 280 mM NaCl,1.5 mM Na_2_HPO_4_, pH 6.96) and incubated for 15–20 min at room temperature. The final solution was added dropwise to each well. For tau seeding experiments, 1 μM of K19 tau fibrils (based on the molecular mass of K19) were directly added to the cell media an hour after transfection (Xia et al., 2019). 16 h after transfection, cells were washed with PBS and grown in 3% FBS. Cells were harvested at 48 h after transfection.

### 2.5. Cell-based tau aggregation assay

HEK293T cells were lysed in 200 μL of Triton Lysis Buffer (25 mM Tris–HCl, pH 7.5, 150 mM NaCl, 1 mM EDTA, 1% Triton X-100, 20 mM NaF) with a cocktail of protease inhibitors (Waxman and Giasson, 2011; Xia et al., 2019). Cell lysates were centrifuged at 100,000 g and 4°C for 30 min to isolate soluble and insoluble fractions. The insoluble fractions were washed, centrifuged again at 100,000 g and 4°C for 30 min, and resuspended in Triton Lysis Buffer. SDS sample loading buffer (10 mM Tris, pH 6.8, 1 mM EDTA, 40 mM DTT, 0.005% bromophenol blue, 0.0025% pyronin yellow, 1% SDS, 10% sucrose) was added to the cellular fractions that were heated at 95°C for 10 min. The insoluble fraction was sonicated and heated at 95°C for another 10 min to completely dissolve the pellets. The percentage of aggregated tau was calculated as pellet/ (supernatant + pellet) * 100.

### 2.6. Cell-based MT binding assay

HEK293T cells were lysed in 200 μL of PEM buffer (80 mM PIPES, pH 6.8, 1 mM EGTA, 1 mM MgCl_2_) supplemented with 0.1% Triton X-100, 2 mM GTP, 20 μM Paclitaxel, and protease inhibitors (Vogelsberg-Ragaglia et al., 2000; Xia et al., 2019). Cell lysates were heated at 37°C for 30 min and centrifuged at 100,000 g for 30 min. The supernatant was separated from the pellet, and the pellet fraction (MT fraction with bound proteins) was resuspended and homogenized in PEM buffer. SDS sample loading buffer was added to both fractions. Equal amounts of supernatant and pellet were loaded for immunoblotting. Percentage of MT-bound tau was calculated as pellet/ (supernatant + pellet) * 100.

### 2.7. Western blotting

Equal proportions of each sample were loaded on 10% polyacrylamide gels and resolved by gel electrophoresis. After electrophoretic transfer to nitrocellulose membranes, the blots were blocked in 5% milk with Tris-buffered saline for an hour. For phosphorylation-specific antibodies, the blots were blocked in 5% bovine serum albumin (BSA). Primary antibody was added and incubated overnight at 4°C at 1: 1000 dilution for 3026 tau antibody (Strang et al., 2017; Xia et al., 2021a) or β-tubulin antibody (clone TUB 2.1, Fisher Scientific, Waltham, MA). Phosphorylation-specific tau antibodies 7F2 and AT180 were used to detect tau phosphorylated at Thr205 and Thr231, respectively (Goedert et al., 1994; Strang et al., 2017). The next day, the samples were incubated in goat anti-rabbit or anti-mouse secondary antibodies conjugated to horseradish peroxidase (Jackson Immuno Research labs, Westgrove, PA) for an hour. After TBS washes, the membranes were exposed and imaged using Western Lightning Plus ECL reagents (PerkinElmer, Waltham, MA). Each immunoblot image was imported into ImageJ program (National Institutes of Health, Bethesda, MD) for densitometric analysis. Statistical analysis was performed with Graphpad Prism software (San Diego, CA) and one way ANOVA with Dunnett’s test was used to calculate group comparisons.

### 2.8. Immunofluorescence and Thioflavin S staining

For immunofluorescence, HEK293T cells were washed in PBS and fixed in 4% paraformaldehyde for 10 min. Autofluorescence eliminator reagent (Millipore, Burlington, MA) was added for five minutes and washed with 40% ethanol. Under dark conditions, slides with cells were incubated in 0.0125% Thioflavin S dissolved in 50% ethanol/ PBS for 3 min and washed in 50% ethanol and PBS. Slides were submerged in blocking solution (2% FBS/0.1% Triton-X-100 in PBS) for 30 min. Primary antibody in 2% FBS/PBS was incubated for one hour. After PBS washes, Alexa-fluor 594 conjugated anti-rabbit antibody (Invitrogen, Carlsbad, CA) were added at 1: 500 dilution for one hour. Slides were washed in PBS and placed in 0.5 μg/mg of 4′,6-diamidino-2-phenylindole (DAPI, Invitrogen, Carlsbad, CA) in PBS for 5 min. The coverslips were mounted using Fluoromount-G (Invitrogen, Carlsbad, CA). Fluorescent images were captured with a BZ-X700 Keyence digital microscope (Itasca, Il).

For quantification, different 20X fields of ~20–50 cells were captured for each treatment group using a BZ-X700 Keyence digital microscope (Itasca, Il). Tau positive cells that colocalized with Thioflavin S positive aggregates were calculated as a ratio to total number of tau positive cells and reported as a percentage of Thioflavin S positivity.

## 3. Results

### 3.1. MT binding properties of 0N3R tau are differentially affected by missense tau mutations located around the fourth repeat

**Figure 2:**
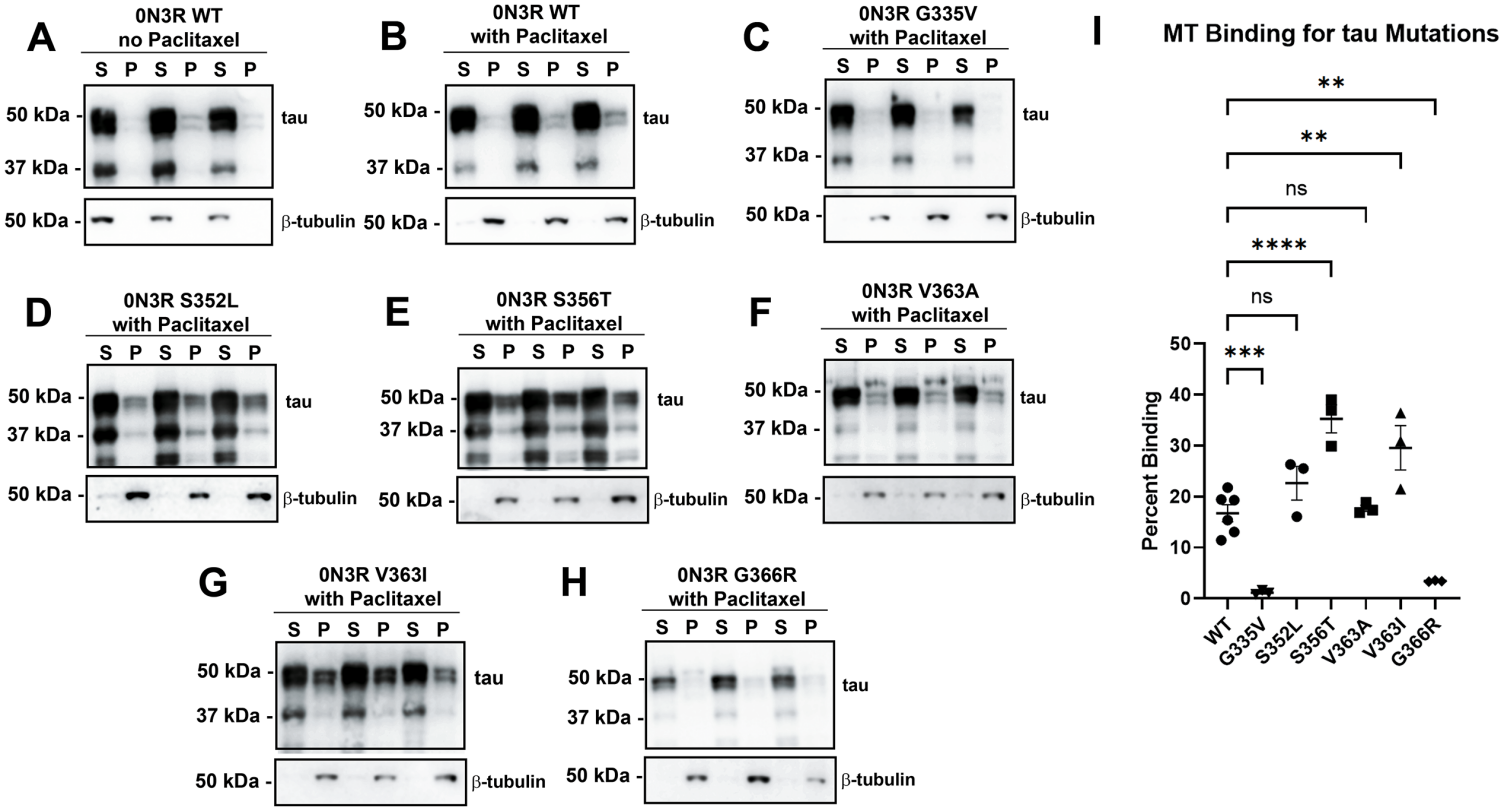
Tau mutations within or near the fourth MT binding repeat of tau have differential effects on tau-MT interactions. HEK293T cells were transfected to express WT tau in the 0N3R isoform and were **(A)** untreated or **(B)** treated with Paclitaxel in a cell-based MT binding assay. In the presence of Paclitaxel, the same assay was used on other tau mutations in the 0N3R isoform including **(C)** G335V, **(D)** S352L, **(E)** S356T, **(F)** V363A, **(G)** V363I, and **(H)** G366R. S = soluble and P = pellet. The immunoblots were probed with antibodies against β-tubulin (clone TUB 2.1) and for total tau (3026 antibody). The relative mobilities of molecular weight markers are shown on the left. **(I)** One-way ANOVA with Dunnett’s Test was performed with N = 6 for WT tau and N = 3 for tau mutations. **** = *p* < 0.0001, *** = *p* < 0.001, ** = *p* < 0.01, and ns = not statistically significant.

We previously showed that many missense tau mutations in the 0N4R isoforms generally displayed reduced MT binding when compared to WT tau (Xia et al., 2019). To expand upon these results, we investigated a series of pathogenic tau mutations clustered around the R4 MT repeat within the 0N3R isoforms (Figure 1). In this assay, the drug Paclitaxel is used to stabilize MT assembly and proteins that associate with MTs can be isolated by high-speed centrifugation (Vallee, 1982; Xia et al., 2019). In the absence of Paclitaxel, most of the tubulin remains in the soluble fraction (Figure 2A). In the presence of Paclitaxel, the majority of tubulin is polymerized into MTs and is found in the pellet fraction (Figure 2B). Relative to WT 0N3R tau, G335V and G366R 0N3R tau displayed reduced MT binding, while S356T and V363I 0N3R tau were more highly associated with MTs (Figure 2). The MT binding of S352L and V363A 0N3R tau did not significantly differ from WT 0N3R tau.

### 3.2. Different pathogenic tau mutations in the 0N3R isoform show varied aggregation propensity

**Figure 3:**
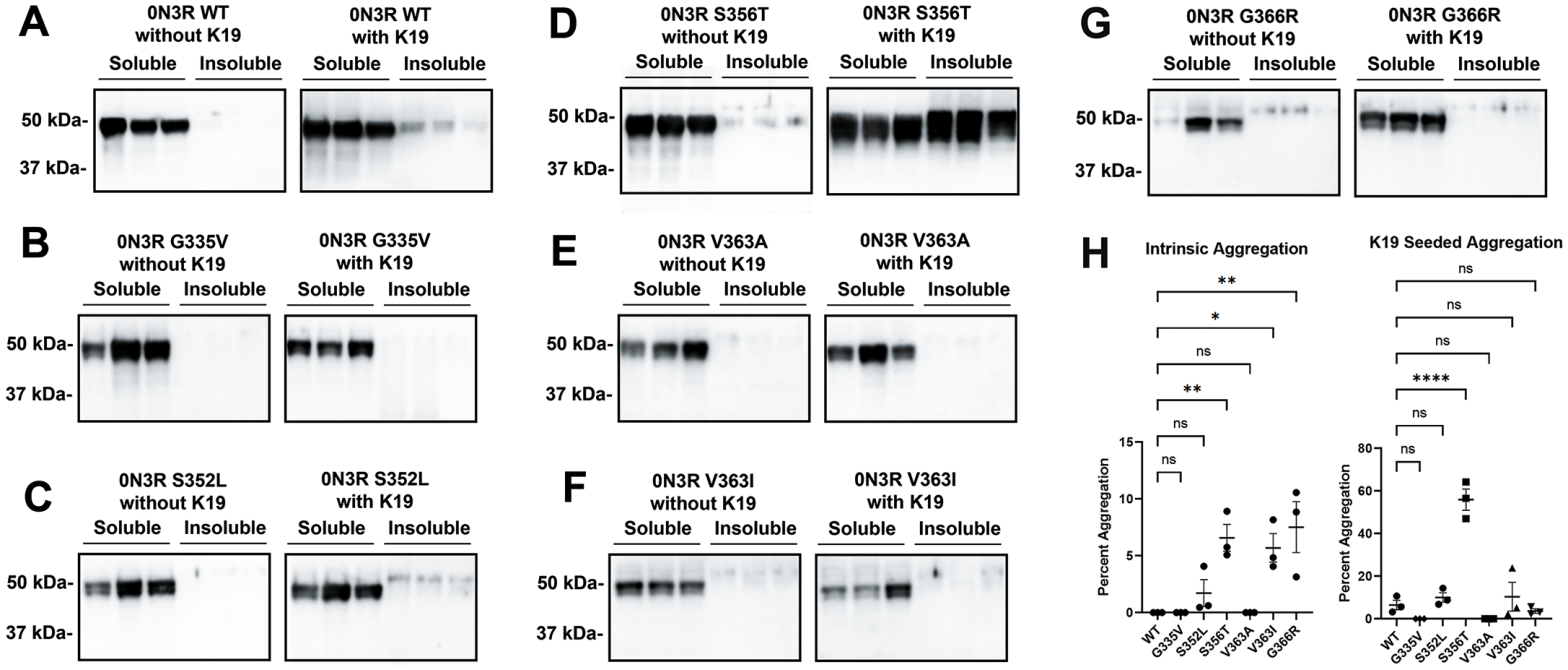
Comparison of tau mutants reveal that within the 0N3R isoform, S356T is uniquely capable of K19 seed-induced aggregation. HEK293T cells were transfected to express either **(A)** WT tau or different tau mutations **(B)** G335V, **(C)** S352L, **(D)** S356T, **(E)** V363A, **(F)** V363I, **(G)**, and G366R and untreated or treated with K19 seeds. Biochemical aggregation assays were performed as described in Materials and Methods and assessed by immunoblotting with tau antibody 3026. The relative mobilities of molecular weight markers are shown on the left. **(H)** Graph shows percent aggregation of 0N3R WT tau and different tau mutations. One-way ANOVA with Dunnett’s Test was performed with N = 3 for WT tau and all mutations. **** = *p* < 0.001, ** = *p* < 0.01, * = *p* < 0.05, and ns = not statistically significant.

Previously our group showed that a subset of FTD-causal tau mutations in the 0N4R isoform displayed slight increases in intrinsic tau aggregation; however, most tau mutations did not significantly aggregate (Strang et al., 2018; Xia et al., 2019). To explore isoform-dependent differences, we also expressed different 0N3R tau mutations clustered around the R4 repeat in HEK293T cells. Cells expressing tau variants were either untreated or treated with polymerized K19 tau fibrils, which correspond to the core of the MT binding domain in 3R isoforms (Gustke et al., 1994). The WT 0N3R tau did not intrinsically aggregate without K19 and modestly aggregates with the addition of preformed K19 seeds (Figure 3A). The tau mutants G335V, S352L and V363A did not significantly aggregate with or without K19 seeds (Figures 3B,C,E). In contrast, V363I and G366R have modest intrinsic aggregation but that was not increased by exogenous K19 seeds (Figures 3F,G). S356T 0N3R tau did not intrinsically aggregate but uniquely displayed robust aggregation when cells were treated with K19 seeds (Figures 3D,H). S356T 0N3R tau after K19 seeding shows different phosphorylation patterns in soluble and insoluble fractions (Supplementary Figure S1). Phosphorylation specific antibody 7F2 against pThr205 detected tau in both fractions, but AT180 antibody against pThr231 only showed significant signal in the soluble fraction.

### 3.3. S356T 0N3R tau forms robust prion-like induced aggregates after K19 seeding

**Figure 4:**
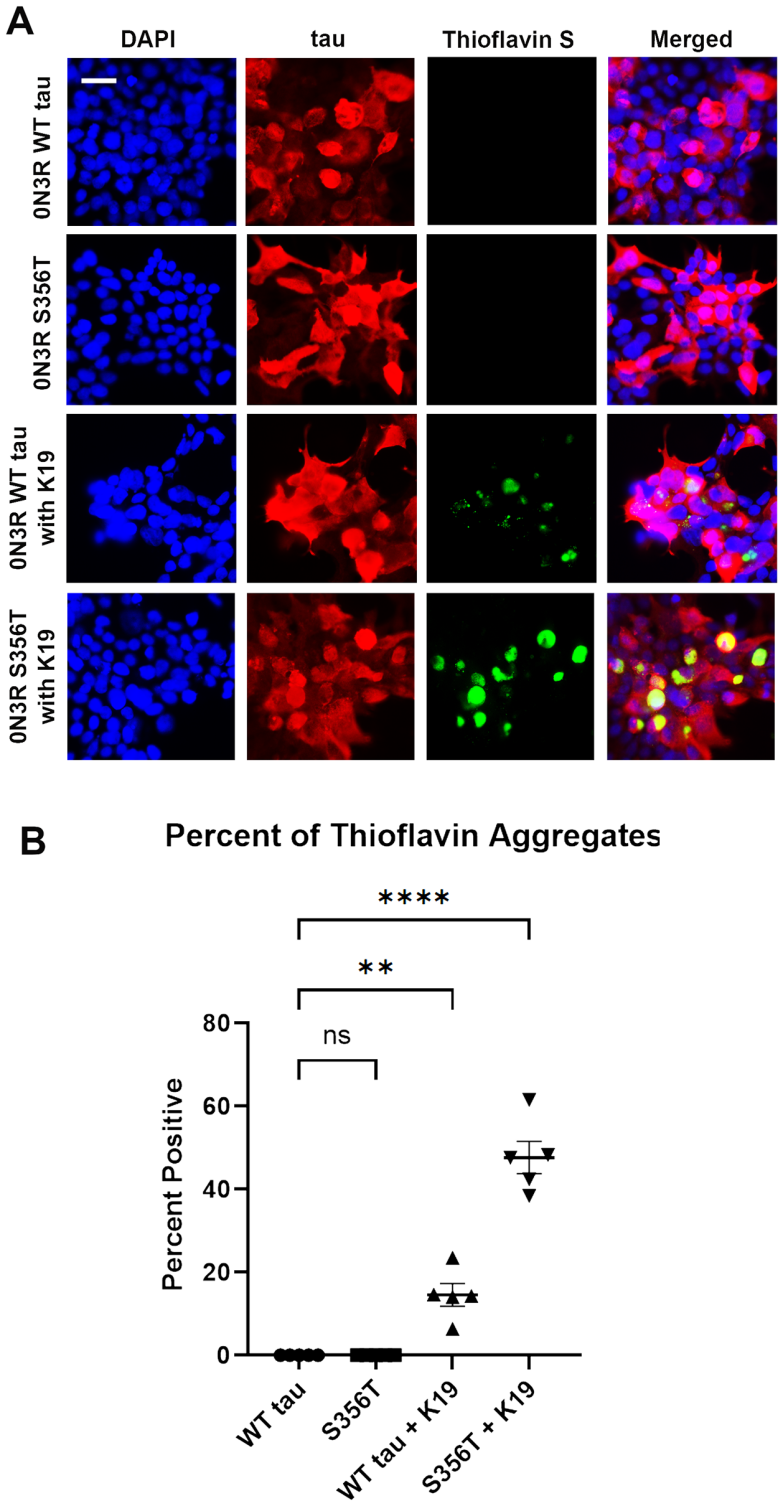
S356T tau is more prone to form Thioflavin S-positive cellular aggregates compared to WT tau from cellular treatment with K19 seeds **(A)** HEK293T cells were transfected to express WT tau or S356T 0N3R tau and untreated to treated with K19 tau fibrils. The samples were stained for immunofluorescence with DAPI for nuclei (blue), tau antibody 3026 (red), and Thioflavin S for protein aggregates (green). Scale bar = 50 μm. **(B)** Graph shows comparison of significant Thioflavin S positive aggregates between different treatment groups. One-way ANOVA with Dunnett’s Test was performed with N = 6 for WT tau and S356T. **** = *p* < 0.001, ** = *p* < 0.01, ns = not statistically significant.

To further assess tau aggregate formation, HEK293T cells were stained with Thioflavin S, which specifically binds to amyloid structures of protein aggregates (Guntern et al., 1992). Using immunofluorescence, HEK293T cells transfected to express WT 0N3R tau and S356T 0N3R tau were stained for total tau expression and Thioflavin S (Figure 4). Without treatment, WT 0N3R tau and S356T 0N3R tau both did not display Thioflavin positive aggregates. Treatment of cells expressing WT 0N3R tau with K19 preformed fibrils resulted in some cellular aggregates and speckles (Figure 4A). By contrast, treatment of cells expressing S356T 0N3R tau with preformed K19 fibrils resulted in larger and more abundant Thioflavin positive cellular tau aggregates (Figure 4).

## 4. Discussion

Previously, our group surveyed tau mutations in the 4R tau isoforms for properties of MT binding and tau aggregation (Strang et al., 2018; Xia et al., 2019). However, many tauopathies also have 3R isoforms that contribute to the formation of tau pathological inclusions (Zhang et al., 2022). To further investigate the effects within 3R tau isoform, we characterized several tau mutations that cause familial forms of FTD and are centered around the R4 repeat that forms the core of tau filaments (Fitzpatrick et al., 2017).

In terms of MT binding and regulation, both 3R and 4R tau isoforms have distinctively different physiologic roles (Goedert and Jakes, 1990; Goode et al., 2000; Panda et al., 2003). Previously, we have demonstrated that most tau mutations in the 0N4R isoform led to decreased MT binding with a few exceptions (Xia et al., 2019). In this study, we further characterized multiple tau mutations within the MT binding region in the context of 0N3R tau isoform. The impact of these tau mutants was more varied: depending on the tau mutation, 3R isoform could lead to increased binding, decreased binding, or no significant change (Table 1). These findings suggest that the effects of 3R tau on MT regulation may have more structural complexity and multiple contributing factors. This outcome has important implications for many tauopathies including AD and FTD, which are characterized by tau aggregates that form from a mix of 3R and 4R tau isoforms (Zhang et al., 2022). Tau is an intrinsically disordered protein that has been shown to demonstrate liquid–liquid separation (Boyko and Surewicz, 2022). The relative balance of 3R and 4R isoforms contribute to tau intermolecular interactions, affect the formation of tau aggregates, and even cause neurodegeneration due to differential splicing (D’Souza et al., 1999; Liu and Gong, 2008).

**Table 1:** Summary of MT binding and tau aggregation for different tau mutations in the 0N3R and 0N4R isoforms for *in vivo* and cell-based studies.

Tau mutations	0N4R MT binding	0N3R MT binding	0N4R Tau aggregation	0N3R Tau aggregation
G335V	↓ (Xia et al., 2019)	↓*	↑ (Xia et al., 2019)	↔*
S352L	↓ (Xia et al., 2019)	↔*	↔ (Xia et al., 2019)	↔*
S356T	↓ (Xia et al., 2019)	↑*	↑ (Xia et al., 2019)	↑↑*
V363I	↓ (Xia et al., 2019)	↑*	↑ (Morelli et al., 2018; Xia et al., 2019)	↑*
V363A	↓ (Xia et al., 2019)	↔*	↔ or ↑ (Morelli et al., 2018; Xia et al., 2019)	↔*
G366R	↓ (Xia et al., 2019)	↓*	↔ (Xia et al., 2019)	↑*

Asterisk (*) represents results of the current study.

Most of the 0N3R tau mutations near the R4 repeat do not significantly aggregate with or without preformed K19 tau fibrils. Unexpectedly, S356T 0N3R tau display robust propensity K19 seeding induced aggregation compared to WT 0N3R tau. This effect is not seen in the 0N4R isoform of S356T, which was resistant to prion-like aggregation by K18 seeds (Xia et al., 2019). The tau mutation S356T is an incredibly aggressive form of early onset FTD that presents in the mid to late 20s with psychotic features similar to schizophrenia (Momeni et al., 2010; Khan et al., 2012). It was predicted that S356T lead to Pick’s disease, a FTD subtype that is primarily a 3R repeat tauopathy (Momeni et al., 2010). Is it likely that 0N3R S356T tau represents a distinctive prion-type strain that is prone to robust seed-induced aggregation on levels comparable to Pro301 mutations in the 4R isoforms (Strang et al., 2018; Xia et al., 2019). This was confirmed with extensive formation of Thioflavin positive aggregates in 0N3R S356T tau compared to WT tau after K19 seed induction.

While the point mutation from serine to threonine in S356T is a relatively minor change, the additional methyl group leads to increased nonpolar interactions that could accelerate tau filament formation. In particular, the R4 repeat of tau is an area within the core of tau filaments (Fitzpatrick et al., 2017; Falcon et al., 2018). This amino acid change may have led to increased tau-tau interactions that is further accelerated by K19 seeds. Another potential explanation for the aggregation propensity of S356T is the alteration in regulation of different post-translational modifications such as phosphorylation. After K19 seeding, 0N3R S356T tau showed different phosphorylation patterns in soluble and insoluble fractions. Tau in the soluble fraction was reactive for 7F2 antibody against tau pThr205 and AT180 antibody against tau pThr231, while the insoluble fraction was only positive for the 7F2 antibody (Supplementary Figure S1). Therefore, it is possible that the S356T mutation may alter specificity to cellular kinases or phosphatase. Ser356 is a particularly important tau phosphorylation site that is elevated in post-mortem analysis of tau filaments isolated from AD brains (Morishima-Kawashima et al., 1995; Hanger et al., 2002; Xia et al., 2021c). Multiple tau kinases regulate Ser356 phosphorylation including GSk-3β, MARK, and Nuak1 (Meyer et al., 1995; Wang et al., 1998; Lasagna-Reeves et al., 2016). Deletion of Nuak1 kinase and a decrease in Ser356 phosphorylation improved memory deficits and overall phenotype of P301S transgenic mice (Lasagna-Reeves et al., 2016), which supports the importance of pSer356 as an important phosphorylation site. Ser356 is also located within the highly conserved KXGS motif, which is close to an important K353 acetylation site that is protective against tau aggregation (Cook et al., 2014; Xia et al., 2021a). It is also possible that the S356T mutation may allosterically hinder K353 acetylation.

In conclusion, 3R tau isoform display increased mutant selective variation in MT binding ability depending on the region within the MTBD. While most tau mutations in the 0N3R isoform did not aggregate, our data revealed that the 0N3R isoform of S356T tau is robustly vulnerable to prion-like seeded aggregation. This new finding will lead to better modeling and elucidate the contribution of 3R tau isoforms to the diverse presentation of different tauopathies.

## Data availability statement

The raw data supporting the conclusions of this article will be made available by the authors, without undue reservation.

## Author contributions

YX, BB, and JK performed experiments. YX and BG co-wrote the manuscript. All authors contributed to the article and approved the submitted version.

## Funding

This work was supported by the University of Florida. YX was supported by fellowship F30AG067673 from the National Institute on Aging.

## Conflict of interest

The authors declare that the research was conducted in the absence of any commercial or financial relationships that could be construed as a potential conflict of interest.

## Publisher’s note

All claims expressed in this article are solely those of the authors and do not necessarily represent those of their affiliated organizations, or those of the publisher, the editors and the reviewers. Any product that may be evaluated in this article, or claim that may be made by its manufacturer, is not guaranteed or endorsed by the publisher.
