# Supplementary material for: Tau mutation S356T in the three repeat isoform leads to microtubule dysfunction and promotes prion-like seeded aggregation
Source: Front Neurosci. 2023 May 25;17:1181804. doi: 10.3389/fnins.2023.1181804 (PMC10248064; doi:10.3389/fnins.2023.1181804)
Supplement: Supplementary file 1 [file Image_1.pdf]

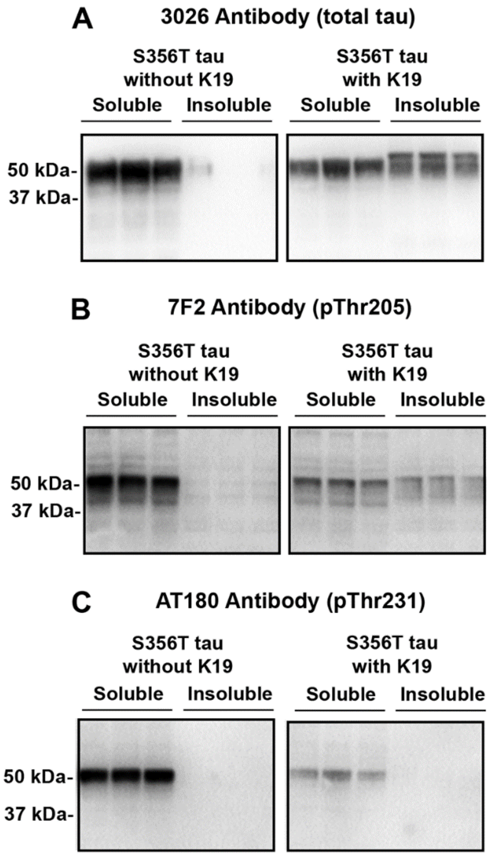


**Supplemental Figure 1**. **0N3R S356T tau has different phosphorylation patterns in soluble and insoluble aggregated fractions.** HEK293T cells transfected to express 0N3R S356T tau were untreated or treated with K19 fibrillar seeds. Biochemical aggregation assays were performed as described in Materials and Methods and assessed by immunoblotting with (**A**) 3026 antibody against total tau (**B**) 7F2 antibody against tau phosphorylated at Thr205 or (**C**) AT180 antibody against tau phosphorylated at Thr231. The relative mobilities of molecular weight markers are shown on the left.
